# Adaptive Forecasting of Nuclear Power Plant Operational Status Under Sensor Concept Drift Using a Bridging Distribution Adaptive Network

**DOI:** 10.3390/s24227241

**Published:** 2024-11-13

**Authors:** Kui Xu, Linyu Liu, Yang Lan, Shuan He, Huajian Fang, Minmin Cheng

**Affiliations:** China Nuclear Power Operation Technology Corporation, Wuhan 430233, China; liuly01@cnnp.com.cn (L.L.); lanyang@cnnp.com.cn (Y.L.); heshuan@cnnp.com.cn (S.H.); fanghj01@cnnp.com.cn (H.F.); chengmm@cnnp.com.cn (M.C.)

**Keywords:** nuclear power operation, sensors, adaptive bridge distribution, concept drift

## Abstract

A large number of sensors are required to collect information during the operation of nuclear power plants to ensure their absolutely safe operation. However, because of the unique nature of nuclear reactions, the physical environment of nuclear power production is prone to changes, leading to concept drift in the data collected by the sensors. Concept drift describes the phenomenon of sample distribution changing over time, which typically negatively impacts the model’s training and inference processes. We found that nongradual distribution changes could be guided by generating transitional intermediary distributions within the distribution, thereby achieving a gradual change process. Based on this, we designed a bridging distribution adaptive network (BDAN), which consisted of identical-depth TDoA (time difference of arrival) homomorphic backbone neural networks on both sides with a latent adaptive bridging module in the middle. By calculating the distribution differences over multiple timesteps, a series of bridge distributions were generated to guide the gradients in the latent space, updating the parameters of the latent adaptive guiding module in a directional manner and enabling the model to perceive nongradual distribution changes in the time domain. Experimental results showed that the BDAN outperformed the previous state-of-the-art benchmark methods by 5.6% in terms of mean squared error in the nuclear power data prediction task under concept drift, achieving the best fault prediction performance.

## 1. Introduction

Solving the problem of concept drift in nuclear power monitoring data is a difficult challenge for time-series modeling. When a reactor in a nuclear power plant starts working, the temperature and pressure of the core dissolution change rapidly, triggering changes in the distribution of multiple sensor data. When the external environment changes significantly, the reactor control system also receives different degrees of impact. Therefore, the nuclear power plant needs to quickly and accurately adjust the model according to the changing patterns in the sensor data [1] and predict the subsequent operating conditions. The bridging distribution adaptive network can provide a solution to this problem. It is a neural network model for modeling time-series data [2] that uses a set of bridging functions to represent the underlying probability distribution of time-series data [3].

In a BDAN, a set of bridging functions is first used to transform the time-series data into a set of probability density functions (PDFs). Bridge functions model the conditional distribution of time-series data given past values and estimate the PDFs of time-series data at different points in time. Once the PDFs are estimated, they are used to train the BDAN. The training of the BDAN can be divided into two phases. In the first stage, the network is trained using the monitoring data of the nuclear power plant to learn the initial parameters of the bridge distribution. In the second stage, with the emergence of new data, the network is gradually updated so that it can adapt to changes in the statistical properties of the time series. The advantage of the BDAN is that it can deal with gradual and abrupt changes in time series at the same time, which is suitable for forecasting in dynamic environments such as nuclear power production [4,5,6,7]. In addition, BDANs can deal with missing data and noisy input problems that are common in real-world time-series prediction problems [8]. The BDAN is an effective method to solve the concept drift of nuclear power monitoring data, and its effectiveness in different fields of application needs to be further studied. In machine learning models, usually only one time period of data can be input into the model for training, and each input period is assumed to be identically distributed. However, in reality, the local data distribution of time series changes over time, leading to concept drift in the time series [9]. The popularity of the Internet of Things and the high variability of the observable environment have amplified the occurrence of concept drift, posing greater challenges to the analysis of time-series data. In the past, it was a common practice to detect drift in time series and, when concept drift occurred, retrain the model to fit the new distribution using the latest generated data. Although these methods have achieved a certain degree of adaptive concept drift, they rely too much on the latest data [10] and have difficulty capturing patterns when there are sporadic or seasonal changes in the data [11].

**Figure 1:**
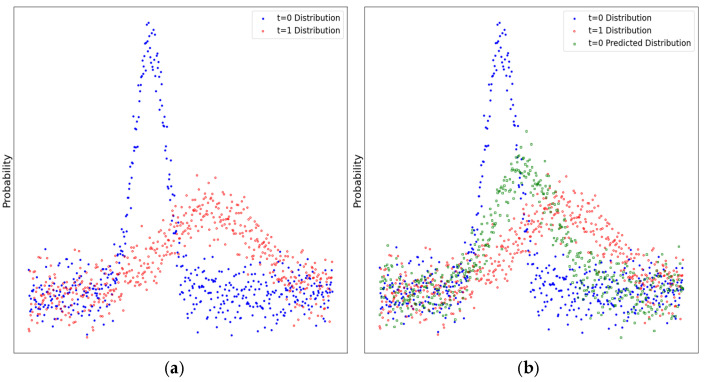
Prediction of concept drift using a BDAN. (**a**) illustrates the change in data distribution from time t = 0 to time t = 1. (**b**) displays the data distribution at time t = 1 predicted by the BDAN based on the known distribution at t = 0, which closely aligned with the actual distribution at t = 1.

We propose the bridge distribution adaptive network for recognizing patterns and making predictions in time-series data that experience concept drift. The BDAN’s backbone consists of two symmetric “encoder–decoder” structures connected by an adaptive bridge module that includes a series of alternating noisy inputs. This module’s encoders transform the input series of white noise into a set of bridge distributions based on the latent distribution of the data at the current and next timesteps. Figure 1a illustrates the change in data distribution from time t = 0 to time t = 1. Figure 1b displays the data distribution at time t = 1 predicted by a BDAN based on the known distribution at t = 0, which closely aligns with the actual distribution at t = 1. The main contributions of this study are as follows:

- First, to address the issue of distribution differences caused by concept drift in nuclear power plant sensor data, we propose an adaptive bridge module. This module generates multiple sequences of bridge distributions based on the distribution differences before and after the drift. These bridge distributions serve as intermediaries between the two distributions, guiding the time-series encoder to transition more smoothly to the postdrift distribution.
- Second, to address the issue of missing prediction paths in the model, our proposed multilayer bridge distribution structure allows the BDAN to bridge any finite-scale distribution differences. Once the first bridge distribution is generated, subsequent bridge distributions can be infinitely decomposed, thus providing a predictable path for monitoring data before and after the concept drift.
- Last, to handle the new distributions emerging from sensors, we introduce a sampling layer based on the bridge distribution. This layer encodes and samples the bridge distribution, and the decoded information is no longer a deterministic latent representation but a random variable that follows the bridge distribution. This enables the BDAN to perform more creative reconstruction of the predicted samples and enhances the model’s generalization capabilities for new emerging distributions.

The content of this study is divided into five parts. The first part is the introduction, which presents an overview of this research. Section 2 covers related work, providing a comprehensive overview of the latest advancements in the fields of data prediction and concept drift, as well as related areas relevant to this study. Section 3 introduces our proposed model, explaining the theory and technical principles of the BDAN, from theoretical analysis to structural design. Section 4 focuses on the experimental part, where the effectiveness and superiority of the BDAN were validated through model performance evaluation, ablation studies, and parameter analysis. Finally, we conclude the paper with a summary of the entire work.

## 2. Related Work

The concept drift accompanying the nuclear power production process has attracted more and more attention from scholars, and it exists in various forms [6]. Based on quantitative measurement methods for the rates of concept drift and seasonality [7], various types of concept drift can be measured, as they are closely linked to the implicit patterns present in sequence data [8], and our research is based on the latent patterns of historical and current data to generate bridge distributions in concept drift. Previous studies focused on predicting concept drift [12,13] by predicting the trend of changes in the input data distribution to make adjustments to the model in advance. However, in most cases, such adjustments are not accurate and may hinder the learning of the model. Our method does not require predicting future concept drift but needs only to fill the gap in data distribution between the current and next timesteps, which allows the model to extract patterns in relatively smooth distribution changes.

Based on the model training approach, concept drift can be categorized as incremental training [14] and retraining [15]. The former focuses on the impact of new data on the overall distribution and attempts to find clues to concept drift from the overall data change pattern. The latter focuses on the impact of new data on the current window data and no longer pays attention to the pattern of historical data. Our proposed method combines the advantages of these two methods. On the one hand, the BDAN utilizes historical and current data to train the model, fully utilizing the seasonal rules in historical data. On the other hand, the BDAN generates adaptive bridge distributions using current and next timestep data to mitigate the challenges brought by distribution changes.

Self-attention mechanisms have achieved remarkable success in representing both sequence and image information patterns [16]. Because of their better long-range memory effect on sequence information, various variants of transformer-based models have been heavily used in time-series modeling, with related studies focusing on long-range prediction of time series [17,18,19]. However, the original transformer model had several drawbacks when dealing with time series, including its time complexity of O(*n*^2^) and tendency to trigger error accumulation. Later variants are still based on the self-attention mechanism with reduced time complexity, which alleviates the error accumulation problem [20,21]. These methods make full use of the pattern recognition ability of the self-attention mechanism to extract the patterns in the latent distribution of time series and try to represent the distribution differences caused by concept drift. The BDAN is a time-series prediction method different from the self-attention mechanism. It can not only predict time-series values but address the challenge of concept drift during training. While transformer-based variants have strong long-range memory, this memory can sometimes be a drawback when dealing with concept drift. This is because concept drift may generate distributions that have never been observed before. The BDAN actively resolves this issue, making it a promising alternative for time-series prediction in scenarios where concept drift is likely to occur.

As research into graph neural networks (GNNs) deepens, a variety of time-series analysis and prediction schemes based on graph embedding have been proposed recently [22,23]. The use of graph neural networks to analyze multisource data with complex feature associations has demonstrated significant representational advantages. For instance, when the data stream exhibits prominent spatiotemporal characteristics, graph neural networks can effectively embed data features and their spatiotemporal correlations [24,25], making them suitable for traffic flow prediction. In fact, when there are inherent connections between sensors, graph neural networks can adeptly recognize patterns in the high-dimensional features they constitute.

Several strategies have been proposed for coping with concept drift based on domain generalization [26,27]. Since concept drift can be regarded as a change in the time domain, domain-adaptive and generalization methods can also be employed for concept drift representation learning. Among them are data-augmentation-based methods and data generation methods, which intervene in the input data by randomizing and generating different samples, thus improving the model’s pattern adaptation and generalization capabilities. In contrast, domain-invariant learning [28] aligns feature distributions by means of adversarial training to learn the domain invariance embedded in changing patterns. In addition to the aforementioned methods, feature-decoupling-based approaches [29] have been proposed to improve model generalization by separating the common parts of the domain. Another class of domain generalization methods is based on model training strategies [10], which aim to enhance the model’s adaptive ability. These strategies include gradient methods and metalearning methods [30].

Although these methods apply domain-adaptive and generalization techniques to concept drift, they are static and do not adapt to situations where the data distribution changes significantly before and after concept drift. In contrast, the BDAN is a dynamic method that adjusts the encoder’s output in real time based on the postdrift distribution, guiding the model output towards the postdrift scores. In order to overcome the problems in existing works, and to resolve the data distribution problem in time series, this paper is proposing a BDAN-based concept drift mechanism for time-series-based neural networks.

## 3. Proposed Methods

In the operational status prediction task for conventional power generation, sensor data are sampled in a standard manner, and the model uses fixed-length subsequent samples to supervise the training of earlier samples. This self-supervised learning paradigm can produce relatively effective predictive models. However, nuclear power significantly differs from conventional energy. The internal state of a reactor is more extreme than that of conventional energy sources, resulting in lower data distribution stability. Traditional models heavily rely on the assumption of independent and identically distributed (IID) samples, making the monitoring quality of nuclear power production more sensitive to the concept drift phenomenon.

Considering the characteristics of nuclear power production, we have found that using samples spaced at different lengths in the future direction to train the model can force the model to adapt to nongradual changes in sensor data distribution. When the model can detect the trend of sudden changes in this distribution, its ability to adapt to concept drift improves.

We further propose the BDAN, which captures potential patterns of concept change through sensor monitoring data deployed around nuclear power production. This network generates bridging distributions to align the latent patterns before drift with those after drift. The monitoring data returned by the current sensors is guided in the latent space to form possible future distribution patterns, thereby predicting changes in data distribution after the occurrence of concept drift.

### 3.1. Theoretical Framework

The bridging distribution is a transitional distribution between the latent distributions of current and future data, and it is closer to the predicted distribution at a future timepoint when constructed at the present time. To obtain this bridging distribution, the BDAN needs to infer based on current data and build a latent adaptive bridge module in the latent space to adapt to the future distribution. The latent adaptive bridge module is a neural network component that generates the bridging distribution by injecting a set of white noise data and narrowing the distance between the distributions at consecutive timepoints. To obtain this bridging distribution, we first need to extract the patterns and latent distributions from the raw data, which requires pretraining the encoder through parameter updates, using it as a pattern extractor. For the current input data, we aim to obtain the neural network parameters Θ, enabling its latent distribution to output the next sequence value with the highest probability, as shown in Equation (1):

(1) $$\left\{x_{h:t}\right\}\overset{p\left(x_{h:t},\Theta \right)}{\to }\left\{x_{h+1:t+1}\right\}$$

where {*x_h_*_:*t*_} represents the data from time *h* to *t*. In order to obtain $\underset{\Theta }{\arg\max\;}p\left(x_{h:t},\Theta \right)$, we start with the log-likelihood of $p\left(x_{h:t},\Theta \right)$ and perform concept drift adaptive inference on it. According to the properties of definite integrals [10], we have:

(2) $$\begin{array}{l} \log p\left(x_{h:t},\Theta \right)=\int_{z_{c}}q\left(z_{c}|x_{h:t},\theta_{e}\right)\log p\left(x_{h+1:t+1},\theta_{d}\right)dz_{c}\\ \theta_{e},\theta_{d}\subset \Theta \end{array}$$

In (2), Θ represents the set of parameters in the entire model, $\theta_{e},\theta_{d}\subset \Theta$ are the parameters, *q* represents any probability, and *z_c_* is the integration variable. Further derivation yields:

(3) $$\begin{array}{l} \int_{z_{c}}q\left(z_{c}|x_{h:t},\theta_{e}\right)\log p\left(x_{h+1:t+1},\theta_{d}\right)dz_{c}\\ =\int_{z_{c}}q\left(z_{c}|x_{h:t},\theta_{e}\right)\log\left[p\left(z_{c},x_{h+1:t+1},\theta_{d}\right)/p\left(z_{c}|x_{h+1:t+1},\theta_{d}\right)\right]dz_{c}\\ =\int_{z_{c}}q\left(z_{c}|x_{h:t},\theta_{e}\right)\log\left[\frac{p\left(z_{c},x_{h+1:t+1},\theta_{d}\right)}{q\left(z_{c}|x_{h:t},\theta_{e}\right)}\cdot \frac{q\left(z_{c}|x_{h:t},\theta_{e}\right)}{p\left(z_{c}|x_{h+1:t+1},\theta_{d}\right)}\right]dz_{c}\\ =\underset{term1}{\underbrace{\int_{z_{c}}q\left(z_{c}|x_{h:t},\theta_{e}\right)\log p\left[\frac{p\left(z_{c},x_{h+1:t+1},\theta_{d}\right)}{q\left(z_{c}|x_{h:t},\theta_{e}\right)}\right]dz_{c}}}+\\ \underset{term2}{\underbrace{\int_{z_{c}}q\left(z_{c}|x_{h:t},\theta_{e}\right)\log\left[\frac{q\left(z_{c}|x_{h:t},\theta_{e}\right)}{p\left(z_{c}|x_{h+1:t+1},\theta_{d}\right)}\right]dz_{c}}} \end{array}$$

In (3), the second term is the Kullback–Leibler divergence, and since the Kullback–Leibler divergence is always greater than or equal to 0, the first term becomes a lower bound on $\log p\left(x_{h:t},\Theta \right)$. Maximizing this log-likelihood function is equivalent to maximizing this lower bound. Therefore, we can discard the second term and focus on the derivation of the first term.

(4) $$\begin{array}{l} \underset{term1}{\underbrace{\int_{z_{c}}q\left(z_{c}|x_{h:t},\theta_{e}\right)\log p\left[\frac{p\left(z_{c},x_{h+1:t+1},\theta_{d}\right)}{q\left(z_{c}|x_{h:t},\theta_{e}\right)}\right]dz_{c}}}\\ =\int_{z}q\left(z_{c}|x_{h:t},\theta_{e}\right)\log p\left[\frac{p\left(x_{h+1:t+1}|z_{c},\theta_{d}\right)p\left(z_{c}|\theta_{d}\right)}{q\left(z_{c}|x_{h:t},\theta_{e}\right)}\right]dz\\ =-D_{KL}\left[q\left(z_{c}|x_{h:t},\theta_{e}\right)||p\left(z_{c}|\theta_{d}\right)\right]+E_{z_{c}∼q\left(z_{c}|x_{h:t},\theta_{e}\right)}\left[p\left(x_{h+1:t+1}|z_{c},\theta_{d}\right)\right] \end{array}$$

In (4), the expression in the second line can be decomposed using Bayes’ theorem into a KL divergence term and an expectation term. *D_KL_* represents the Kullback–Leibler divergence. Maximizing (4) during the pretraining phase forces the encoder, with input{*x_h:t_*}, to output a latent distribution *z_c_* and make its sampling under the influence of the decoder parameters *θ_d_*, output the next timestep data {*x_h_*_+1:*t*+1_} with maximum likelihood. Minimizing *D_KL_* forces the latent distribution generated by the encoder to follow a Gaussian distribution as closely as possible.

The self-supervised signal for current data {*x_h:t_*} in the pretraining phase comes from the next samples {*x_h_*_+1:*t*+1_}, during which the parameters *θ_e_* in the encoder are updated, and the encoder becomes a pattern extractor encoding towards the future. It outputs a latent representation *z_c_* in the latent space. In the formal training phase, the bridge module, which is a symmetric structure with white noise vectors injected into the middle region, is enabled. Then, after being mapped by neural networks on both sides, the white noise is transformed into a latent distribution that is similar to the current latent distribution *r_c_* and the next latent distribution *r_n_*. This transformation process is defined as:

(5) $$b_{1}\left(r_{c},r_{n}\right)=\min\left\{{\left\|r_{c}-g_{c}\left(\varepsilon \right)\right\|}_{2}^{2}+{\left\|g_{n}\left(\varepsilon \right)-r_{n}\right\|}_{2}^{2}\right\}$$

where $b_{1}\left(r_{c},r_{n}\right)$ represents the bridge distribution with respect to *r_c_* and *r_n_*, and *g_c_* and *g_n_* represent the encoding functions of the left and right neural networks L-NN and R-NN, respectively, in the bridge module for ε. Equation (5) minimizes the distance between ε and the latent representations on both sides, thus transforming ε into a bridge distribution. During formal training, the BDAN replaces the original information transmission path with the path generating the bridge distribution, incorporating the bridge distribution into the encoding process and guiding the current input towards generating a latent distribution in the direction of concept drift.

After concept drift occurs, the degree of mutation in the input data distribution may become very large. In this case, a single bridge distribution may not be sufficient to bridge the gap between distributions. The BDAN can use the first generated bridge distribution to split into multiple bridge distributions.

(6) $$\begin{array}{l} b_{2c}\left[r_{c},b_{1}{\left(r_{c},r_{n}\right)}_{n}\right]\\ =\min\left\{{\left\|r_{c}-g_{c}\left(\varepsilon_{2}\right)\right\|}_{2}^{2}+{\left\|g_{n}\left(\varepsilon_{2}\right)-b_{1}\left(r_{c},r_{n}\right)\right\|}_{2}^{2}\right\} \end{array}$$

(7) $$\begin{array}{l} b_{2n}\left[b_{1}\left(r_{c},r_{n}\right),r_{n}\right]\\ =\min\left\{{\left\|b_{1}\left(r_{c},r_{n}\right)-g_{n}\left(\varepsilon_{2}\right)\right\|}_{2}^{2}+{\left\|g_{n}\left(\varepsilon_{2}\right)-r_{n}\right\|}_{2}^{2}\right\} \end{array}$$

*b*_2*c*_ and *b*_2*n*_ represent the bridge distributions that are split for the second time. In fact, (6) and (7) provide a recursive structure, where *b*_2*c*_ and *b*_2*n*_ serve as the splitting centers and new bridge distributions can be further split from them. For instance, *b*_2*c*_ can be split into two new bridge distributions:

(8) $$b_{3c}\left(r_{c},b_{2c}\right)=\min\left\{{\left\|r_{c}-g_{c}\left(\varepsilon_{3}\right)\right\|}_{2}^{2}+{\left\|g_{c}\left(\varepsilon_{3}\right)-b_{2c}\right\|}_{2}^{2}\right\}$$

(9) $$b_{3c}\left(b_{2c},b_{1}\right)=\min\left\{{\left\|b_{2c}-g_{c}\left(\varepsilon_{3}\right)\right\|}_{2}^{2}+{\left\|g_{c}\left(\varepsilon_{3}\right)-b_{1}\right\|}_{2}^{2}\right\}$$

In (8) and (9), *b*_2*n*_ can be split into $b_{3n}\left(r_{n},b_{2n}\right)$ and $b_{3n}\left(b_{2n},b_{1}\right)$. The number of splits in the bridge distribution can be set as needed. The distributions obtained after multiple splits are weighted and averaged, and the resulting distribution is passed to the neural network at the current time as probability distribution parameters to generate the imported bridge distribution density function.

### 3.2. Sampling of the Latent Bridge Distribution

The BDAN replaces the latent distribution generated by pretraining with a latent bridge distribution, so sampling is performed based on the bridge distribution. The bridge distribution itself is also a latent representation vector, which is again encoded by the *NN* to output a set of parameters from a multivariate Gaussian distribution, thereby generating a specific density function at the sampling level:

(10) $$\left\{\begin{array}{l} \left(\mu ,\sum \right)=E\left(b_{i}\right)\\ s\sim N\left(\mu ,\sum \right) \end{array}\right.$$

In (10), *μ* represents the mean function of a multivariate Gaussian distribution, Σ represents the covariance matrix, *E* represents the mapping of the neural network *NN*, *s* represents the sampling, and N represents the Gaussian distribution. In the pretraining and formal training phases, the supervised signal is the data for the next timestep, and the parameters in the decoder decode the sampling *s* in the future direction.

Whereas *s* is derived from the bridge distribution, the decoder does not face the large distribution differences arising from concept drift when reducing *s*, allowing for enhanced data reconstruction and prediction performance. Ultimately, the loss function of the BDAN can be formalized as

(11) $$\begin{array}{l} Loss=-D_{KL}\left[q\left(r_{c}|x_{h:t},\theta_{e}\right)||p\left(r_{c}|\theta_{d}\right)\right]+\\ E_{z_{c}\sim q\left(r_{c}|x_{h:t},\theta_{e}\right)}\left[p\left(x_{h+1:t+1}|s,\theta_{d}\right)\right] \end{array}$$

Unlike in (4), in (11), the latent representation at the encoder side is changed from *z_c_* to *r_c_*, and the input at the decoder side is replaced by *s*, reflecting the role of bridge distribution intervention.

### 3.3. Overall Structure

**Figure 2:**
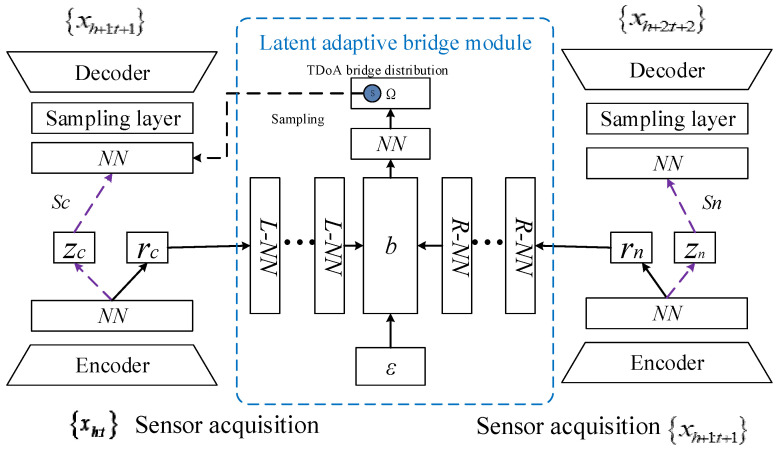
BDAN overview.

In order to implement the calculation process of Equation (4), we designed a BDAN as a symmetric TDoA (time difference of arrival) structure, as shown in Figure 2, the two sides of which were composed of TDoA homograph backbone neural networks of the same depth. The TDoA homomorphic backbone neural network received the monitoring samples of the current and next timesteps and performed feature extraction and reconstruction on them. The middle part was the latent adaptive bridge module, which was built in the latent space and calculated the difference between the distribution of samples passed by the backbone network after multiple timesteps and the current distribution. This was designed to guide the gradient in the latent space to update the network parameters, so that the model could perceive the nongradual change of the distribution over longer distances.

The information flow in the pretraining phase is represented by the purple dashed line. The data are mapped to the latent space through the encoder and the fully connected neural network to form the latent representation zc. Then, through the sampling operation, a latent sample representation Sc is formed, and the decoder reconstructs Sc into a distribution estimate for the next timestep.

In the formal training phase, the information flow is represented by the black solid line. The BDAN encodes the input samples at the current and next timesteps into the current latent representation rc and the next latent representation rn, respectively. The difference between them and the white noise distribution ε in ABM is minimized according to Equations (6) and (7), and *ε* is transformed into a series of latent bridge distributions. Because the middle bridge distribution b carries the same amount of distribution information before and after concept shift, the BDAN takes this distribution as the intermediate state and transmits it to the neural network to map to a set of normal distribution parameters. Thus, the TDoA bridge distribution associated with the intermediate states is generated. The BDAN samples the TDoA bridge distribution to obtain a sample s, which is passed into the decoder for sample reconstruction after concept drift. The loss function in the training phase of the whole neural network is performed as in Equation (11).

## 4. Experiments and Discussion

Unlike in conventional power plants, because of higher safety requirements, the spatial correlation of multisource data generated during nuclear power production is relatively weak, but the correlation in the time domain is stronger. This is determined by the extreme physical environment of the reactor, where various sensors are set to be highly sensitive to indicators related to the chain reaction, resulting in lower stability of the monitoring data distribution. The experiment consisted of three parts. The first part tested the BDAN’s prediction performance under concept drift conditions; the second part evaluated the change in model performance after removing various components of the BDAN through ablation experiments, indirectly demonstrating the contribution of each component; and the third part analyzed the impact of hyperparameters on model performance by altering the BDAN’s key hyperparameters.

The steps of the experiment were as follows: First, the experimental data came from the reactor units and corresponding machinery of a nuclear power plant. The sensor equipment included the The sensor equipment included the nuclear power mechanical system ACP1000 (manufactured by China National Nuclear Corporation, Beijing, China), voltage measurement device DN20 (supplied by Shanghai Electric Group, Shanghai, China), rectifier bridge stack KBPC5010 (provided by Vishay Intertechnology, Malvern, PA, USA), electric pump meter YTP100ML MF (manufactured by Yokogawa Electric Corporation, Tokyo, Japan), and flow measurement device SQL-2500 (supplied by Siemens AG, Munich, Germany). They collected data on pressure, temperature (Temp), water level (WL), reactor total power (TRP), and emergency injection flow (flow). Using the RELAP5 [31] accident analysis program, concept drift in the data was located, and the data were time-aligned and assembled into a multivariate time series, forming a time-series dataset containing 15% concept drift intervals. The dataset was divided into 67,510 batches, with each batch containing 80% training data and 20% test data.

These data were fed into the BDAN, which output the predicted data for the next timestep. The model’s prediction performance was evaluated by measuring the gap between the predicted and actual values.

The experiment’s CPU was an Intel i7-12700, and the GPU was an NVIDIA RTX3090 with 24 GB of memory. The system memory capacity was 32 GB. The deep learning framework used was PyTorch 1.7.1. During the experiment, NVIDIA CUDA was enabled, so the BDAN’s deep learning computations were handled by the GPU.

The baseline models included VAE [11], GRU [30], LSTM [32], Informer [33], and ODE [34]. VAE, or variational autoencoder, uses a set of encoders and decoders for supervised or self-supervised learning. Because of the sampling process introduced between the encoder and decoder, the decoder outputs reconstructed samples that follow the probability distribution of the input data. This gives the model a certain generative capability, allowing it to effectively handle changes in data distribution. LSTM and GRU are improvements to recurrent neural networks (RNNs) that mitigate the gradient vanishing and exploding problems by adding gating units. Informer is a neural network model based on the self-attention mechanism, which has strong long-term memory capabilities. The ODE method is based on neural ordinary differential equations and is used to fit latent distributions. It can handle incomplete input data and, to some extent, alleviate the concept drift problem. Table 1 illustrates the technical features of these models.

To apply these baseline methods, we adopted the same hyperparameter settings as in the corresponding references. However, when the same baseline involves multiple time-series forecasting subtasks, some studies have used different combinations of hyperparameters to handle the different subtasks, allowing the baseline to achieve the best predictive performance on those subtasks at the time. For fairness, we used the same hyperparameter settings as the baseline for each subtask. We conducted 10 tests for each parameter set on the dataset, and the result with the best average mean squared error (MSE) was selected as the result for the baseline. The MSE was calculated as follows:

(12) $$MSE=\frac{1}{m}\sum_{t=1}^{m}{\left(x_{t}-{\hat{x}}_{t}\right)}^{2}$$

where *m* represents the number of samples, *x_t_* represents the actual sample value at time *t*, and ${\hat{x}}_{t}$ represents the predicted sample value at time *t*. On the other hand, the BDAN adopted a unified hyperparameter setting. The fully connected neural network in the main network was set with two layers, and the default setting for the number of splits in the bridge distribution was two, generating a total of three bridge distributions. The Adam optimizer [35] was used with a learning rate of 0.001 in the experiments.

The parameters that need to be randomly initialized in a BDAN consist of two parts. The first part includes the weights contained in the neural network, and the second part is the white noise distribution ε. In the experiment, we performed 12 random initializations for them and averaged the prediction results, recording the range of standard deviations.

### 4.1. Forecasting Performance

The forecasting performance of the model with the number of splits in the bridge module was set to two, i.e., three adaptive bridge distributions were generated. The performance metric was MSE. The results are shown in Table 2.

As shown in Table 2, the BDAN achieved the best mean squared error (MSE) results in predicting concept drift data. This indicates that the adaptive bridge module effectively overcame the challenges posed by distributional changes. The BDAN, being a general neural network with a VAE structure, outperformed single VAE models in baseline methods, where the VAE structure alone did not significantly improve scores. This was because, in addition to containing a sampling layer, the BDAN incorporated the ABM structure, which allowed it to adapt to distributional changes, thereby providing better generalization performance than the VAE. LSTM and GRU, as gated recurrent neural networks, serve as backbone networks for training data, and while their representational performance is superior to that of BDAN, their lack of modules for adapting to distributional changes causes performance degradation when data distributions shift over time. ODE, with its continuous representational structure, naturally possesses some degree of adaptability to concept drift. However, this structure is not specifically designed to handle sequence distributional changes, and its representational capacity is relatively weak, resulting in comparatively lower model performance.

Informer, with its self-attention mechanism, offers strong long-range representational capabilities. However, the neural network lacks components for capturing distributional changes, meaning it requires a higher degree of independent and identically distributed (IID) data, making it less suitable for monitoring concept drift in nuclear power plants. We performed a *t*-test on the prediction results between the second-best performing model, Informer, and the BDAN, which achieved the best results. The *p*-value was 0.039, indicating a statistically significant difference in model performance.

**Figure 3:**
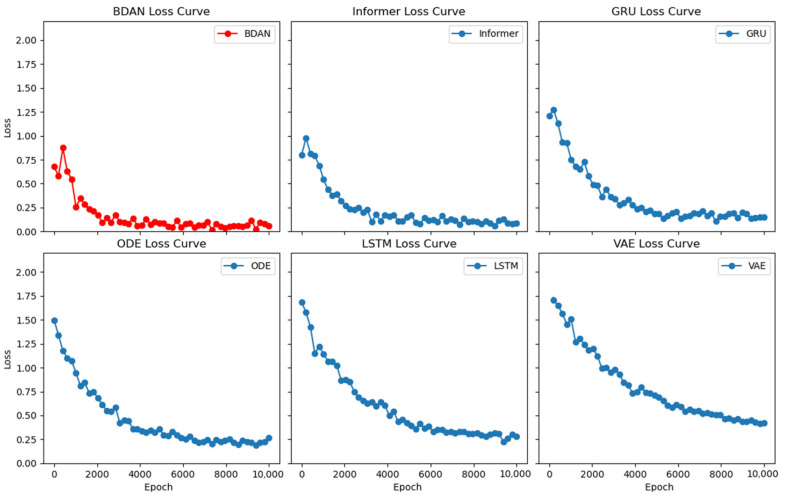
Comparison of convergence.

As shown in Figure 3, compared with the baselines, the BDAN had the fastest convergence rate in the LOSS curve, with Informer following closely behind. Although both exhibited fast convergence efficiency, the BDAN achieved a lower LOSS value. This also indicated that the adaptive bridge distribution module and sampling layer were already playing a role during the training phase. While the BDAN’s backbone network lacked the powerful self-attention representation mechanism of Informer, it still demonstrated better convergence properties in the frequently changing distribution environment of nuclear power plant monitoring, thanks to its stronger generalization capability for concept drift.

### 4.2. Ablation Experiment

In this section, we conducted ablation experiments to investigate the contributions of different components in the BDAN. Specifically, we removed the adaptive bridge module (ABM) and the sampling layer (SL) from the BDAN separately and examined their impacts on the model’s predictive performance measured by mean squared error (MSE). Results are shown in Table 3.

The plus sign indicates keeping the component unchanged, while the minus sign indicates removing the component. The study found that when only the ABM (adaptive bridge module) was removed, the model’s performance significantly declined. When the ABM was retained but the SL (sampling layer) was removed, the model was unable to perform sampling, resulting in a lack of generalization capability. However, the degree of performance decline was not as severe as when the ABM alone was removed. This indicates that both components had a significant impact on model performance, with the ABM having a greater impact. When the ABM was missing, the model struggled to bridge the distributional differences before and after concept drift and lost its adaptive mechanism for handling concept drift, degrading into a simplified version of the VAE structure, leading to a significant performance drop. When the SL structure was missing, the model could no longer perform sampling operations, eliminating its generative capabilities, which also resulted in a decline in reconstruction performance.

**Figure 4:**
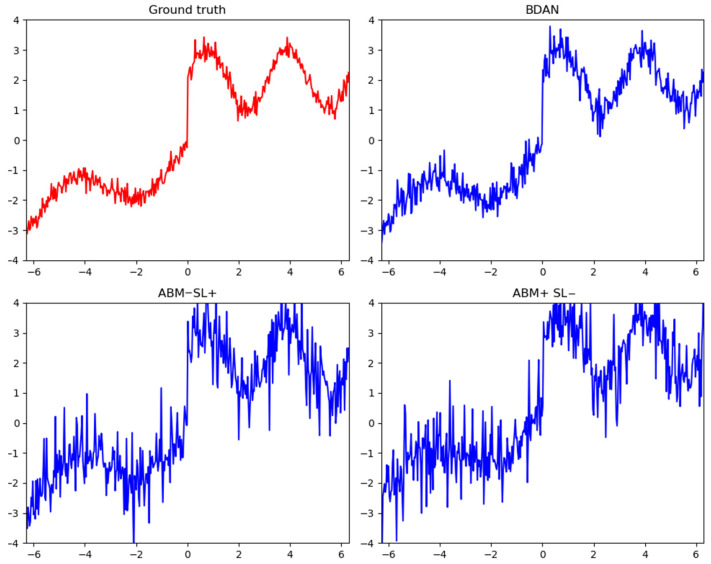
Results of ablation experiments. The red curve in the figure represents the ground truth, while the blue curve represents the reconstruction of the ground truth.

Figure 4 shows the impact of missing components on the model’s predictive ability. When concept drift occurred at timepoint 0, the ground truth reflected the concept drift region within the sample interval, while the other three charts represent the model’s predictions for this region. The BDAN was able to accurately reconstruct the data characteristics before and after the concept drift. However, when the ABM or SL (sampling layer) components were removed, the predicted values showed a significant increase in amplitude, leading to inaccurate sample reconstruction. Especially when the SL is removed, the fluctuations in predicted values before and after the concept drift were large, making the changes in distribution less apparent.

### 4.3. Hyperparameter Analysis

This section analyzes the hyperparameters related to concept drift in the BDAN, namely, the number of bridge distributions generated in the adaptive bridge module. Generally, the more bridge distributions generated, the denser the intermediate distribution connecting the concept drift gap. In Section 4.1 and Section 4.2, we set this value to three. This section presents experiments showing the performance of the BDAN under different numbers of bridge distributions.

**Figure 5:**
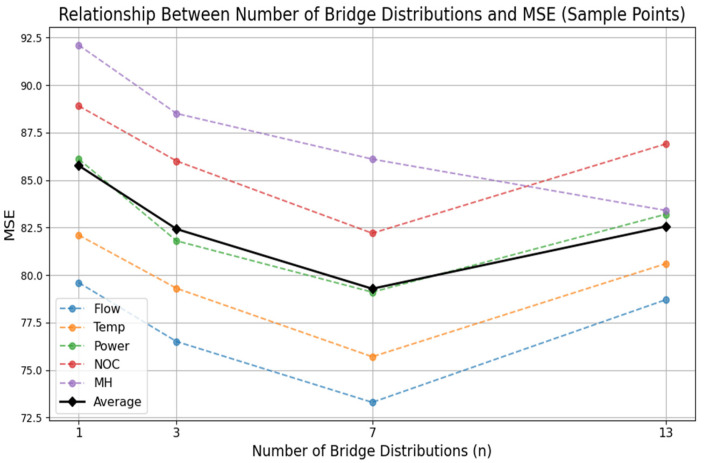
The impact of hyperparameter *n* on model performance.

As shown in Table 4 and Figure 5, *n* represents the number of bridge distributions split out. From the results, the model decreased when the number of bridge distributions decreased to 1 or increased to 13 overall, but the predictive performance further improved when the number was appropriately increased to 7. This suggests that the number of bridge distributions needs to be within an appropriate range. When the number of bridge distributions was 13, the model performed best on the MH dataset, which had many concept drifts occurring in the form of distribution mutations. The results showed that increasing the number of bridging distribution modules could alleviate the distribution difference in data in the time domain to a certain extent and enhance the prediction performance of the BDAN.

### 4.4. Discussion

The experimental results of this study showed that the BDAN demonstrated significant advantages in handling concept drift data, particularly excelling in the mean squared error (MSE) metric. As seen in Table 1, the BDAN maintained a high level of predictive accuracy in environments with concept drift. This advantage is attributed to its unique adaptive bridge module (ABM), which dynamically captures changes in data distribution and addresses the distribution shift problem that traditional methods struggle to handle.

A comparison with other commonly used methods further highlighted BDAN’s advantages. Recurrent neural networks (RNNs) such as LSTM and GRU typically outperform the BDAN in representational power because of their strong time-series modeling capabilities. However, these models lack mechanisms to adapt to changes in data distribution over time, leading to a significant drop in performance in concept drift environments. In contrast, the BDAN not only relies on VAE structures for sampling but adjusts adaptively to changes in data distribution through the ABM module, significantly improving its generalization performance.

ODE (ordinary differential equation networks), as a model based on continuous dynamic systems, is inherently capable of handling smoothly changing time series. However, its representational capacity is relatively weak, and its design is not specifically targeted at dealing with distribution shifts. As a result, it did not perform as robustly as the BDAN in addressing complex concept drift problems. Although ODE has an advantage in capturing continuous changes, it cannot effectively handle sudden distributional shifts, which is particularly evident in environments like nuclear power plants.

The Informer model’s self-attention mechanism enhances its ability to capture long-range dependencies, making it highly advantageous in handling long-sequence data. However, Informer relies on the assumption of independent and identically distributed (IID) data, and its neural network architecture lacks specialized components for dealing with distribution drift. Therefore, in cases where data distribution changes dynamically, Informer’s performance is somewhat lacking compared with the BDAN’s adaptive adjustment capabilities. A *t*-test between the BDAN and the second-best-performing Informer yielded a *p*-value of 0.039, indicating a statistically significant difference in performance.

## 5. Conclusions

Predicting the data collected by nuclear power plant sensors is a crucial task for ensuring the safe operation of nuclear power. However, the issue of sensor concept drift has consistently hindered the accuracy of nuclear power data predictions. To address this, we propose the BDAN model, which can adaptively monitor and adjust the underlying distribution of sample data by detecting changing patterns and relationships within the samples, thereby mitigating the negative impact of concept drift on data prediction tasks. By comparing the differences between the current and future latent distributions, the BDAN generates a series of latent bridge distributions. These bridge distribution modules replace the original latent distributions in the backbone network, forcing the model to reference the latent information of future data, thus improving the model’s adaptability to changes in future data distributions. The model can predict future distribution trends based on historical distribution changes, providing early warnings for adverse sudden conditions and enhancing the safety of nuclear power plant operations. Experiments comparing multiple baseline methods validated the effectiveness and superiority of the BDAN in nuclear power data monitoring. However, the BDAN has certain limitations, as the model’s performance is somewhat dependent on the selection of hyperparameters. As demonstrated in Section 4.3, inappropriate parameters can lead to a rapid decline in model performance, making automatic parameter optimization a key focus of our future research.

## Figures and Tables

**Table 1:** Technical features of baselines.

Methods	Comparison of Technical Features
VAE	Have generative capabilities
LSTM	Add gating units
GRU	Mitigate gradient vanishing
ODE	Neural ordinary differential equations
Informer	Self-attention mechanism
BDAN	Bridge distributions mitigate concept drift

**Table 2:** Forecasting performance (MSE as %).

Methods	Pressure	Temp	WL	TRP	FLOW
VAE	112.7 ± 3.3	106.9 ± 6.4	105.3 ± 3.5	95.9 ± 3.9	124.4 ± 7.1
LSTM	97.8 ± 4.1	95.7 ± 1.2	94.6 ± 2.2	87.9 ± 2.8	106.4 ± 1.8
GRU	94.9 ± 3.7	91.2 ± 3.0	96.2 ± 2.7	91.8 ± 2.2	96.7 ± 5.2
ODE	96.5 ± 0.8	99.6 ± 1.4	98.5 ± 3.0	96.1 ± 2.5	104.6 ± 1.2
Informer	79.6 ± 3.8	83.3 ± 1.3	88.0 ± 3.6	92.4 ± 5.7	93.3 ± 2.9
BDAN	76.5 ± 1.5	78.3 ± 0.9	82.8 ± 2.4	86.0 ± 1.6	88.5 ± 2.2

**Table 3:** Ablation (MSE as %).

Component	Pressure	Temp	WL	TRP	FLOW
ABM− SL−	132.9 ± 3.5	126.6 ± 5.1	125.6 ± 4.4	116.3 ± 2.9	166.6 ± 3.2
ABM+ SL−	107.0 ± 2.8	100.4 ± 2.1	107.4 ± 1.7	96.7 ± 2.5	106.5 ± 1.9
ABM− SL+	111.9 ± 3.1	116.0 ± 3.4	113.2 ± 2.6	105.1 ± 1.3	141.8 ± 4.1
ABM+ SL+	76.5 ± 1.5	79.3 ± 0.9	81.8 ± 2.4	86.0 ± 1.6	88.5 ± 2.2

**Table 4:** Hyperparameter analysis (MSE as %).

Values	Flow	Temp	Power	NOC	MH
n = 1	79.6 ± 1.6	82.1 ± 2.1	86.1 ± 2.5	88.9 ± 2.1	92.1 ± 2.7
n = 3	76.5 ± 1.5	79.3 ± 0.9	81.8 ± 2.4	86.0 ± 1.6	88.5 ± 2.2
n = 7	73.3 ± 3.1	75.7 ± 3.0	79.1 ± 3.3	82.2 ± 1.5	86.1 ± 2.5
n = 13	78.7 ± 3.2	80.6 ± 2.8	83.2 ± 2.1	86.9 ± 3.2	83.4 ± 3.1

## Data Availability

The data presented in this study are available on request from the corresponding author.
